# *Vibrio cholerae* integrates interspecies quorum-sensing signals to regulate virulence

**DOI:** 10.1128/mbio.01537-25

**Published:** 2025-07-31

**Authors:** Erick Maosa Bosire, Myfanwy C. Adams, Paulina D. Pavinski Bitar, Shannon G. Murphy, Jung-Ho Shin, Joshua S. Chappie, Tobias Dörr, Craig Altier

**Affiliations:** 1 Department of Population Medicine and Diagnostic Sciences, College of Veterinary Medicine, Cornell University 575043, Ithaca, New York, USA; 2 Department of Molecular Medicine, College of Veterinary Medicine, Cornell University 251815, Ithaca, New York, USA; 3 Weill Institute for Cell and Molecular Biology, Cornell University 5922, Ithaca, New York, USA; 4 USDA-ARS Soil Management and Sugarbeet Research Unit, Crops Research Laboratory, Fort Collins, Colorado, USA; Gulbenkian Institute for Molecular Medicine, Oeiras, Portugal

**Keywords:** diffusible signal factors, virulence, fatty acids

## Abstract

The human gut harbors enormous microbial diversity, which influences the gut’s chemical environment. As the production of virulence factors affects bacterial fitness, *Vibrio cholerae* has adapted to sense the gut chemical environment to selectively activate virulence factors at permissible intestinal niches. Here, we show that diffusible signal factors (DSFs) canonically used by gut proteobacteria for quorum-sensing communication can repress expression of essential ToxT-dependent virulence factors cholera toxin (*ctxAB*) and the toxin co-regulated pilus (*tcpA-F*). Unlike canonical quorum sensing, where DSFs are sensed by two-component systems or cyclic di-GMP-regulated proteins, DSFs target the central AraC-type transcriptional regulator ToxT. The most potent of these molecules is cis-2-hexadecenoic acid, which represses *ctxAB* and *tcpA-F* expression by 84-fold and 12-fold, respectively. We find that the length of the carbon chain, the cis-2 double bond, and the carboxylic-acid terminus play important roles in the efficacy of these repressive chemicals. We further demonstrate that these chemicals interact with ToxT, preventing it from binding its target promoters while also rendering it susceptible to degradation. Our findings suggest that c2HDA targets ToxT’s fatty acid-binding pocket, where high-affinity interactions likely maintain a restrained conformation that represses activity. Collectively, our data show that *V. cholerae* senses interspecies DSF signals to regulate virulence activation, providing an opportunity to design interventions targeting the virulence of this pathogen.

**IMPORTANCE:**

*V. cholerae* continues to cause sporadic pandemics globally. Although cholera is a self-limiting acute disease, severe cases often require the use of antimicrobials in addition to rehydration therapy. Efforts to eradicate cholera have been hampered by the increased incidence of antimicrobial resistance and low vaccine coverage. The gut chemical environment, driven largely by the microbiota, has been shown to play important roles in modulating colonization by enteric pathogens. Pathogens utilize this chemical environment as cues to optimize virulence and survival. Understanding the underlying mechanisms of pathogen-microbiota interactions may therefore provide avenues to develop control measures targeting virulence and survival. Many of the mechanisms of pathogen-microbiota interactions have yet to be fully described. We demonstrate that *V. cholerae* integrates interspecies chemical signals (DSFs) produced by members of the microbiota to regulate the expression of important virulence factors. DSFs potently repress the virulence island master regulator (ToxT), effectively attenuating cholera toxin secretion. As other related long-chain fatty acids have been reported to impair ToxT function, and as DSF producers localize to the intestinal crypts and mucus layer, it is likely that *V. cholerae* employs these signals in a spatial-temporal manner to program the induction and repression of the energy-intensive virulence factors. Our work provides a framework for designing interventions to disrupt this virulence and survival program to control this pathogen.

## INTRODUCTION

Enteric pathogens encounter myriad chemical signals in the complex intestinal milieu, which dictate their virulence and survival (1). Survival and colonization by these pathogens are dependent upon a fine balance between the production of energy-intensive virulence factors and cell replication (2, 3). Thus, successful enteric pathogens integrate a plethora of chemical signals to ensure expression of the virulence arsenal at only permissible sites, as well as to repress these factors to evade the immune system (1).

*V. cholerae* colonization requires the production of the cholera toxin (CT) and the toxin co-regulated pilus (TCP), which are encoded by *ctxAB* and *tcpA-F,* respectively. Both factors are located in the virulence island and are under the control of the central AraC-type transcriptional regulator ToxT (4, 5). ToxR, ToxS, and TcpP proteins coordinately activate the transcription of *toxT* (6–8). *V. cholerae* integrates a variety of environmental factors as cues to control this virulence island at the level of both ToxR and ToxT (4, 9). Bile present in the gut lumen represses the transcription of *ctxAB* and *tcpA-F*, likely to ensure that these genes are expressed only when *V. cholerae* is located in the mucus layer of the intestine (10, 11). Unsaturated long-chain fatty acids such as oleic, arachidonic, and palmitic also inhibit the expression of ToxT-dependent virulence genes through their effects on ToxT (11–13). The small molecule virstatin was also found to repress CT production and TCP synthesis through its effects on ToxT (14, 15). In contrast to fatty acids and bile, bicarbonate present in high concentrations in the epithelium enhances ToxT activity (16)

A rare class of long-chain fatty acids termed diffusible signal factors (DSFs) are employed as quorum-sensing signals in a wide variety of plant, animal, and human pathogens (17, 18). DSFs were first described in *Xanthomonas campestris* and subsequently identified in other species such as *Burkholderia cenocepacia, Xylella fastidiosa, Cronobacter spp, Stenotrophomonas maltophilia,* and *Pseudomonas aeruginosa* (19–22). Although DSFs may be composed of different substituents and carbon chain lengths, the characteristic cis-2 double bond is regarded as essential for their effects, as the *trans* isomers of these compounds have no signaling activity (17). Different bacterial genera produce different DSF signals, but reports indicate that genera are more responsive to the major signals they themselves produce (23, 24). DSF synthesis is dependent on the RpfF enzyme, which functions both as an enoyl-CoA dehydratase and a thioesterase to synthesize the cis-2 double bond. Two mechanisms for sensing DSFs have been proposed. Bacteria of the genera *Xanthomonas, Stenotrophomonas,* and *Xylella*, as examples, contain a conserved two-component system (RpfC/RpfG) that directly senses DSFs (25). DSF sensing in genera such as *Burkholderia* instead occurs through a PAS, GGGEF, and EAL-domain protein RpfR (26). Both mechanisms control cyclic di-GMP turnover and thus possess the ability to exert a vast range of regulatory activities (27). Increasing evidence shows that the DSF-signaling system controls several important physiological processes including virulence, biofilm formation, and antibiotic resistance (27, 28). DSFs are also involved in intra- and inter-species and even interkingdom signaling (20, 29, 30).

In this study, we demonstrate that *V. cholerae* integrates interspecies DSF signals to regulate the expression of CT and the TCP, virulence factors that are essential for colonization. The DSF c2HDA more potently inhibits these virulence factors compared with related fatty acids, providing opportunities to investigate this system for potential therapeutics and control strategies.

## RESULTS

### A quorum-sensing signal with a precise fatty acid structure effectively represses genes encoding the cholera toxin

**Fig 1:**
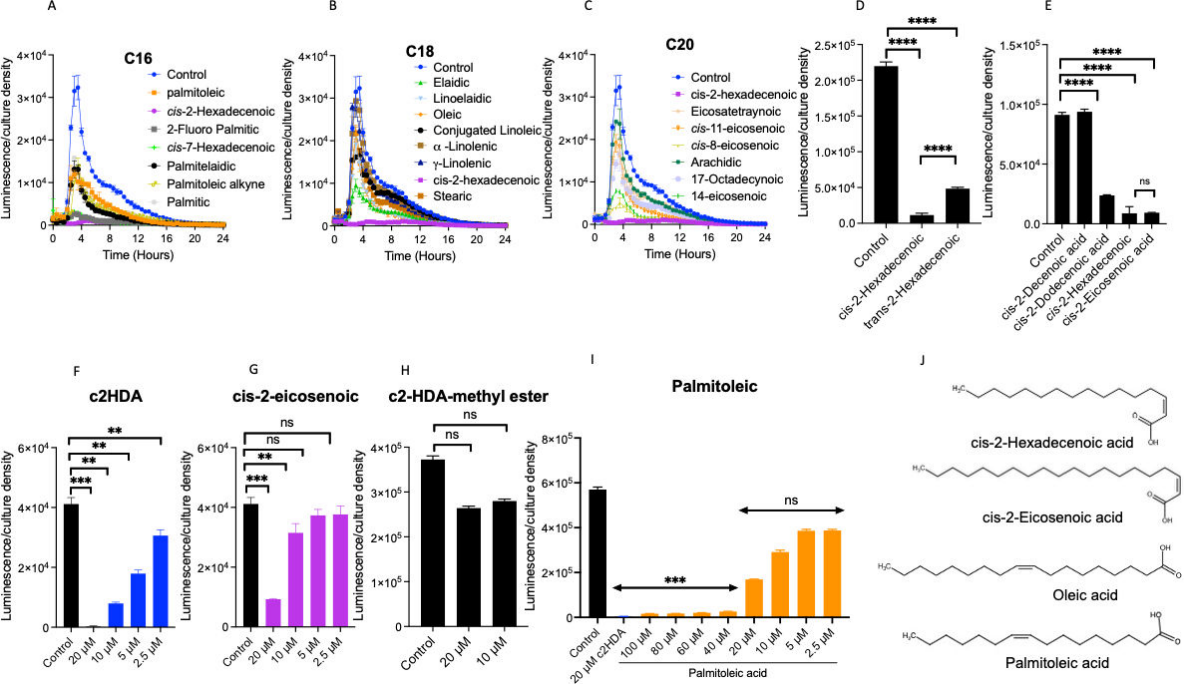
The diffusible signal factor cis-2-hexadecenoic acid (c2HDA) is a highly potent fatty acid signal modulating the expression of virulence genes. A strain carrying a *lacZ::_ctxAB_-luxCDABE* construct was cultured under cholera toxin-inducing conditions in the presence of fatty acids. (**A through C**) Fatty acids with a 16-carbon chain length are overall more potent than those with 18 (**B**) and 20 (**C**) carbons. The reporter strain was cultured in the presence of fatty acids of different chain lengths supplied at 20 µM. Expression of the reporter fusion is presented as luminescence normalized to culture density. (**D**) c2HDA is more potent in repressing *ctxAB* compared to trans-2-hexadecenoic acid. (**E**) c2HDA contains the effective chain length for *ctxAB* repression in comparison to other DSFs. Fatty acids were tested at 20 µM and *ctxAB* expression is presented as peak luminescence normalized to culture density. (**F**, **G**) Dose-dependent response for c2HDA (**F**) and *cis*-2-eicosenoic acid (**G**) at different concentrations as shown. (**H**) The carboxylic-acid terminus is required for the activity of c2HDA. cis-2-hexadecenoic acid methyl ester was tested for its ability to repress *ctxAB* at indicated concentrations. (**I**) Dose-dependent response of palmitoleic acid at different concentrations is indicated in the figure. (**J**) Representative structures of the DSF c2HDA, cis-2-eicosenoic acids, oleic acid, and palmitoleic acid. Expression of the reporter fusion is presented as peak luminescence normalized to culture density. For all the experiments, the control contained the vehicle (DMSO) at identical concentrations with the treated cultures. Error bars represent standard deviations of 5 replicates. Asterisks denote significant differences (****P < 0.0001, ***P < 0.001).

Diffusible signal factors (DSFs) are quorum-sensing molecules derived from long-chain fatty acids that have been shown to mediate interspecies communication (17, 18, 31). As the virulence of *V. cholerae* is known to be affected by long-chain fatty acids through their interaction with ToxT, we sought to determine whether DSFs influence the expression of genes encoding the cholera toxin. We constructed a *lux* reporter-gene fusion to the genes encoding cholera toxin *ctxAB* (*lacZ::_ctxAB_-luxCDABE*). Using this fusion, we found that the quorum-sensing signal with a 16-carbon chain length and a double bond at position 2 (*cis*-2-hexadecenoic acid; c2HDA) was the most repressive, reducing *ctxAB* expression by 84-fold at a concentration of 40 µM (Fig. 1A through C). As c2HDA differed from other 16-carbon fatty acids only in the location of its double bond (Fig. 1J), we hypothesized that the unsaturation at position two in the *cis*-orientation was important for efficacy. To test this, we compared the efficacy of c2HDA with that of its *trans*-isomer, *trans*-2-hexadecenoic acid. c2HDA proved more potent than *trans*-2-hexadecenoic acid, which only reduced *ctxAB* expression by 5-fold (Fig. 1D). These data highlight the importance of the *cis*-orientation, as has been reported for quorum-sensing molecules (17).

We next examined how chain length affected DSF potency in the context of *cis*-unsaturation at position 2 and found that c2HDA and *cis*-2-eicosenoic acid were the most repressive for cholera toxin (CT) gene expression (Fig. 1E). We next compared the efficacy of c2HDA with that of *cis*-2-eicosenoic acid at lower concentrations. At a lower concentration of 10 µM, c2HDA repressed *ctxAB* by 5-fold, whereas *cis*-2-eicosenoic was barely active at this concentration (repressing *ctxAB* by ~8%), indicating that a chain length of 16 carbons was the most effective (Fig. 1F and G). Fatty acids have been previously reported to interact with ToxT by utilizing their carboxylic-acid group to form hydrogen bonds and a salt bridge (11, 13, 32). To determine whether the carboxylic-acid group was important for the functions of c2HDA, we tested the ability of *cis*-2-hexadecenoic acid methyl ester to reduce *ctxAB* expression. We found that disrupting the carboxylic-acid group in this way abolished c2HDA inhibitory effects, indicating that an intact carboxyl-terminus is necessary for its repressive activity on *ctxAB* (Fig. 1H). We confirmed that c2HDA can act as a highly potent inhibitor of virulence signaling in other *V. cholerae* strains, as it was able to repress CT expression similarly in the pandemic strain C6706 carrying a *lacZ::_ctxAB_-luxCDABE* reporter fusion (Fig. S2), indicating that this phenomenon can be generalized among *V. cholerae* strains. Thus, although various fatty acids may inhibit genes encoding the cholera toxin (CT), it appears that a fatty acid signal with a precise chemical structure and functional groups efficiently regulates CT production.

The human gut contains a variety of long-chain fatty acids, including oleic and palmitoleic, often at millimolar concentrations (33). Palmitoleic acid was found to repress the production of virulence factors by *V. cholerae* (12, 13, 32). We sought to determine the minimum concentrations of palmitoleic acid that would repress *ctxAB* to a level comparable with that of c2HDA. We found that 40 µM was required to obtain maximum repression of *ctxAB,* and above this concentration, minimal or no further inhibition was observed (Fig. 1I). Similarly, a concentration of 40 µM was required for oleic acid to maximally repress (Fig. S1B). *cis*-7-hexadecenoic acid produced by some bacteria was only effective at 20 µM. Only the synthetic compound 2-fluoro-palmitic acid, not known to occur in nature, was found to be effective at concentrations lower than 20 µM and showed no dose dependence (Fig. S1A). Thus, common gut fatty acids act only at higher concentrations to inhibit *ctxAB* expression compared with c2HDA.

**Fig 2:**
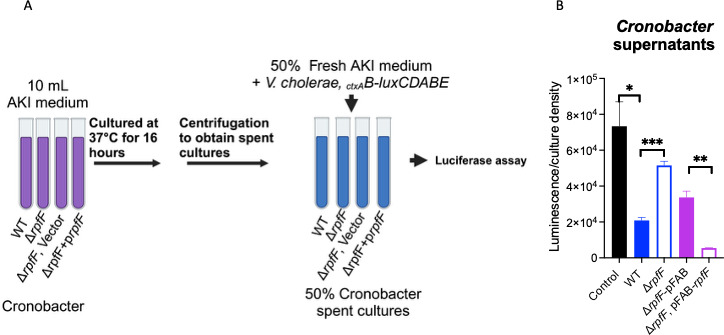
Native concentrations of *Cronobacter* repress *V. cholerae* virulence-gene expression. (**A**) Experimental setup for testing the ability of *Cronobacter sakazakii* spent cultures to repress *V. cholerae* virulence-gene expression. *C. sakazakii* was grown overnight in AKI medium, and supernatants were obtained. Supernatants were diluted with 50% fresh AKI medium, and *V. cholerae* carrying a reporter-gene fusion was introduced. (**B**) *Cronobacter* supernatants repress virulence-gene expression in a *rpfF*-dependent manner. Expression of the reporter fusion is presented as peak luminescence normalized to culture density. Error bars represent standard deviations of 5 replicates. Asterisks denote significant differences (***P < 0.001, **P < 0.01, *P < 0.05).

Unlike most fatty acids, DSFs are expected to be produced in low concentrations. Species capable of producing DSFs have been found in the mucus layer of the small intestines where *V. cholerae* colonizes (34, 35). We sought to determine whether native concentrations of DSFs would repress virulence-gene expression. We cultured *V. cholerae* carrying the *lacZ::_ctxAB_-luxCDABE* construct in spent cultures of *Cronobacter sakazakii,* which is known to harbor an *rpfF* homolog (22). *Cronobacter* was grown in AKI medium overnight, and supernatants were obtained and diluted with 50% fresh medium before inoculation with *V. cholerae* (Fig. 2A). Spent cultures from the wild-type *Cronobacter* repressed *ctxAB* by ~4-fold compared with the Δ*rpfF* mutant, which repressed by ~30% (Fig. 2B). *ctxAB* was repressed ~13-fold by spent cultures of a Δ*rpfF* mutant strain carrying a plasmid with *rpfF* expressed under a constitutive promoter. We speculate that the stronger repression observed under these conditions was most likely due to increased production of the DSF resulting from multicopy *rpfF* expression. Overall, these data indicate that DSF producers repress *V. cholerae* virulence-gene expression in a *rpfF*-dependent manner. Thus, although DSFs are produced at low concentrations, the occurrence of producers in close proximity to *V. cholerae* in the mucus layer may provide a means of physiological interaction.

### c2HDA efficiently represses the transcription of genes encoding cholera toxin, resulting in highly attenuated toxin secretion

**Fig 3:**
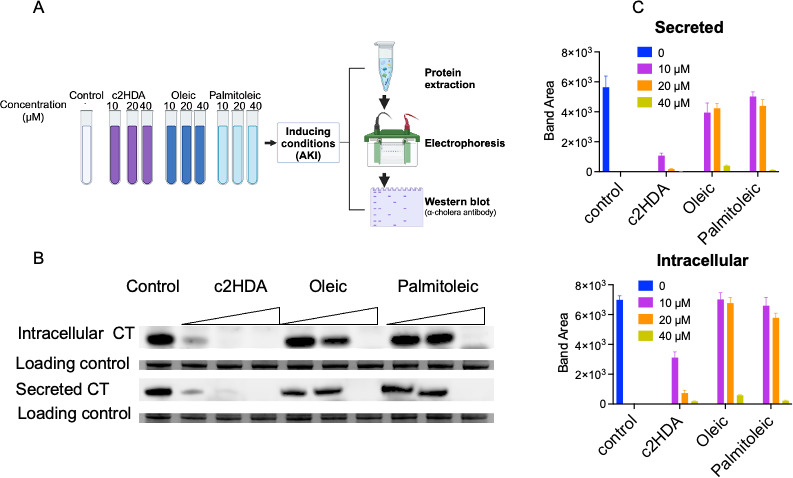
c2HDA mediates reduced cholera toxin production under inducing conditions. (**A**) *V. cholerae* was cultured in AKI medium in the presence of 10, 20, and 40 µM of c2HDA. As controls, oleic and palmitoleic acids were also tested at identical concentrations as c2HDA. (**B**) Western blot detection of intracellular and secreted cholera toxin (CT) levels in the presence of increasing concentrations of fatty acids. (**C**) Area of each toxin band was measured by Image J. The control contained the vehicle (ethanol) at an identical concentration to the treated cultures.

Successful gut colonization by *V. cholerae* is dependent upon the expression of CT and TCP, the expression of both of which is reduced outside the host and in repressing conditions such as laboratory media (36). We anticipated toxin production to be greatly reduced in the presence of a low concentration of c2HDA, as this signal demonstrated a profound efficacy in repressing genes encoding CT, compared with related fatty acids. To test the extent to which c2HDA attenuates CT secretion, we harvested supernatants and bacterial pellets from cultures grown under virulence-inducing conditions and detected the toxin by western blotting using an anti-cholera toxin antibody (Fig. 3A). Cultures were treated with increasing concentrations of c2HDA (10, 20, or 40 µM). As a control, cultures were also treated with fatty acids previously reported to regulate *V. cholerae* virulence-gene expression and are present in the gut (10, 11, 13, 32, 37, 38), such as oleic acid and palmitoleic acid. c2HDA highly attenuated intracellular and secreted CT at the lowest concentration tested (10 µM), whereas the levels of CT in cultures treated with this concentration of oleic acid or palmitoleic acid remained indistinguishable from the untreated control (Fig. 3B and C). Such a high potency for repressing CT production may suggest that c2HDA is acting as a precise signal regulating the pathogenicity of *V. cholerae*.

### c2HDA represses genes encoding the toxin-co-regulated pilus and consequently reduces pilus synthesis

**Fig 4:**
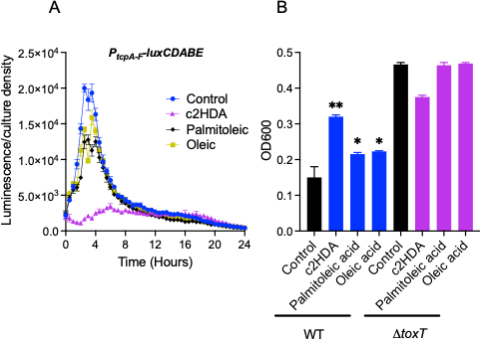
c2HDA represses genes encoding the toxin-co-regulated pilus leading to reduced pilus synthesis. (**A**) A strain carrying a P*_tcpA-F_-luxCDABE* construct was cultured under cholera toxin-inducing conditions in the presence of fatty acids. The chemicals were supplied at 20 µM and expression of *tcpA-F* is presented as luminescence normalized to culture density. Error bars represent standard deviations of 5 replicates. (**B**) c2HDA inhibits agglutination of *V. cholerae*. Cultures were grown in LB medium (pH 6.5) overnight at 30°C. Lower optical density depicts increased agglutination. The control contained the vehicle (ethanol) at an identical concentration to the treated cultures. Error bars represent standard deviations of 5 replicates. Asterisks denote significant differences from the untreated control (**P < 0.01, *P < 0.05).

*V. cholerae* also utilizes the toxin-co-regulated pilus for colonization (39). As the expression of genes encoding both CT and the pilus is controlled through the same regulatory cascade (36), we hypothesized that c2HDA would also reduce the synthesis of the toxin-co-regulated pilus. To test the effects of c2HDA on genes encoding this pilus, we constructed a *luxCDABE* reporter fused to the promoter of genes encoding the pilus *tcpA-F* (P*_tcpA-F_-luxCDABE*). We found that c2HDA reduced *tcpA-F* expression by 12-fold, whereas palmitoleic acid and oleic acid reduced the expression by 2-fold and 1.5-fold, respectively (Fig. 4A). Next, we tested the effects of c2HDA on pilus synthesis by evaluating agglutination of bacteria grown in the presence of this chemical. The toxin-co-regulated pilus plays a role in the formation of micro-colonies, and thus, agglutination has been used to investigate the phenotype of this pilus *in vitro* (9, 40). Cultures were grown overnight at 30°C under established conditions that induce the TCP, and agglutination was examined by estimating the clarity of cultures and formation of aggregates (Fig. 4B). A lower turbidity at OD_600_ signifies increased aggregation in this assay. c2HDA increased culture density by ~2-fold compared with palmitoleic acid, which showed a 69% density increase, both compared with the untreated control. For comparison, as we were testing the activity of the transcriptional activator ToxT on the TCP operon, we also estimated agglutination by a Δ*toxT* mutant. The Δ*toxT* mutant demonstrated the highest turbidity, close to that of the wild type treated with c2HDA, indicating the lowest aggregation. These data demonstrate that c2HDA potently inhibits the expression of genes encoding both CT and the toxin-co-regulated pilus and consequently reduces the production of these colonization factors.

### c2HDA potently represses message levels of genes encoding the cholera toxin and the toxin-co-regulated pilus through ToxT

**Fig 5:**
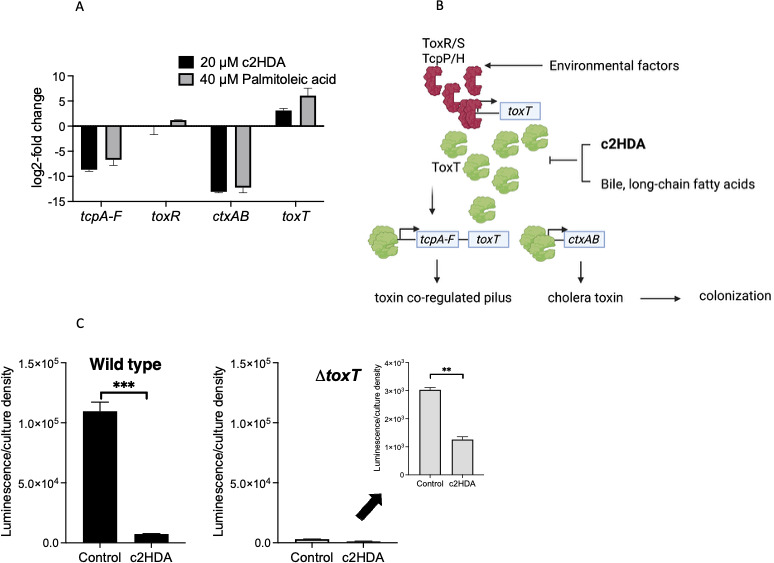
c2HDA acts through ToxT to repress virulence genes. (**A**) c2HDA inhibits the expression of genes under the control of ToxT. Cultures grown under inducing conditions were treated with c2HDA and palmitoleic acid at indicated concentrations, and RNA was extracted for qPCR analysis. Gene expression is presented as log2 fold change. Error bars represent standard deviations of 5 replicates. (**B**) A model for c2HDA’s regulation of *V. cholerae* virulence activation. c2HDA regulates the expression of genes encoding cholera toxin and the toxin co-regulated pilus through the central transcriptional regulator ToxT as reported for other related environmental factors. (**C**) c2HDA primarily inhibits the expression of *ctxAB* through its activity on ToxT. A Δ*toxT* strain carrying a *lacZ::_ctxAB_-luxCDABE* reporter fusion was cultured in AKI medium and supplemented with 20 µM of c2HDA. The insert is the same graph with a lower scale to show the differences between the control and the treated sample. As a control, a wild-type strain was cultured under similar conditions. Expression of *ctxAB* is expressed as peak luminescence per culture density. Error bars represent standard deviations of 5 replicates. Asterisks denote significant differences (***P < 0.001, **P < 0.01).

ToxT is the central transcriptional activator of genes encoding CT and the toxin-co-regulated pilus, and reports have shown that long-chain fatty acids interact with ToxT, disrupting its functions (11, 32). To determine whether c2HDA acts through ToxT, we assessed the effects of c2HDA on the message levels of *toxT* and the virulence genes *ctxAB* and *tcpA-F* that are activated by ToxT. Both *ctxAB* and *tcpA-F* were downregulated by c2HDA, whereas *toxT* was slightly upregulated, indicating that this chemical acts upstream of *ctxAB* and *tcpA-F*, most likely on ToxT which transcriptionally controls these genes, as has been reported for related fatty acids (Fig. 5A). Since *toxT* was slightly upregulated, it is possible that c2HDA may alternatively repress virulence genes by acting on regulators that specifically control ToxT expression. To test this hypothesis, we also determined the effects of c2HDA on the transcription of *toxR,* which is the transcriptional activator of *toxT* in the regulatory cascade. c2HDA had no inhibitory effects on *toxR* message level, demonstrating that c2HDA controls virulence genes post-transcriptionally and/or downstream of this regulator in the cascade and thus affects only those genes under the ToxT transcriptional control (Fig. 5A). As *tcpA-F* is located proximal to *toxT* in the operon, ToxT transcriptional activation of the *tcpA-F* promoter leads to transcription of both *tcpA-F* and *toxT* (Fig. 5B). Thus, ToxT amplifies its own expression under inducing conditions (41). It would thus be expected that with c2HDA treatment, *toxT* message level would be repressed due to perturbation of ToxT. We, however, observed a slight upregulation of *toxT* message, whereas the genes under ToxT control remained repressed, suggesting that this chemical acts at the level of ToxT, in the regulatory cascade, to control *ctxAB* and *tcpA*. To confirm that c2HDA acts through ToxT, we tested the effects of c2HDA in a Δ*toxT* mutant using a *ctxAB::luxCDABE* reporter fusion. As expected, expression of *ctxAB* in the Δ*toxT* mutant was low compared with the wild-type but still sufficient to observe differences between treated samples and controls (Fig. 5C). We found that c2HDA slightly reduced *ctxAB* expression by ~2-fold in a Δ*toxT* mutant compared with ~15-fold in wild-type, indicating that c2HDA might have minor effects on *ctxAB* independent of ToxT. Overall, these data indicate that, like other related fatty acids, c2HDA activity primarily requires ToxT to inhibit the expression of genes encoding CT and the toxin-co-regulated pilus.

Our data implicate that c2HDA is a highly potent signal for controlling genes encoding CT and the toxin co-regulated pilus expression. We thus anticipated a greater reduction of *ctxAB* and *tcpA-F* mRNA by c2HDA, in comparison to related fatty acids. To test the message levels, we treated cultures with either 20 µM c2HDA or 40 µM palmitoleic acid—a concentration that physiologically affects *ctxAB* and *tcpA-F* expression (Fig. 1I and 3B)—and quantified the amount of mRNA produced by qPCR. c2HDA reduced *tcpA* and *ctxAB* mRNA by ~9-fold and 13-fold, respectively, compared with palmitoleic acid tested at a higher concentration, which reduced the mRNA levels of the two genes by ~7-fold and 12-fold, respectively (Fig. 5A).

Our data indicated that c2HDA, like other long-chain fatty acids, acts through ToxT to repress virulence-gene expression. As long-chain fatty acids are known to act directly on ToxT, rather than through their degradation products, we presumed this to be the case with c2HDA as well. To confirm this, we tested the efficacy of the first degradation product of c2HDA in repressing ToxT functions. Fatty acids are metabolized through the β-oxidation pathway in multiple cycles resulting in the removal of two carbons per cycle (42). For c2HDA, the first degradation product would thus be the 14-carbon saturated fatty acid myristic acid. We found that myristic acid had no significant repressive effects on *ctxAB* expression (Fig. S3), indicating that c2HDA acts directly to inhibit virulence-gene expression and not through its degradation products.

### c2HDA reduces ToxT binding to promoter DNA and accelerates its degradation

**Fig 6:**
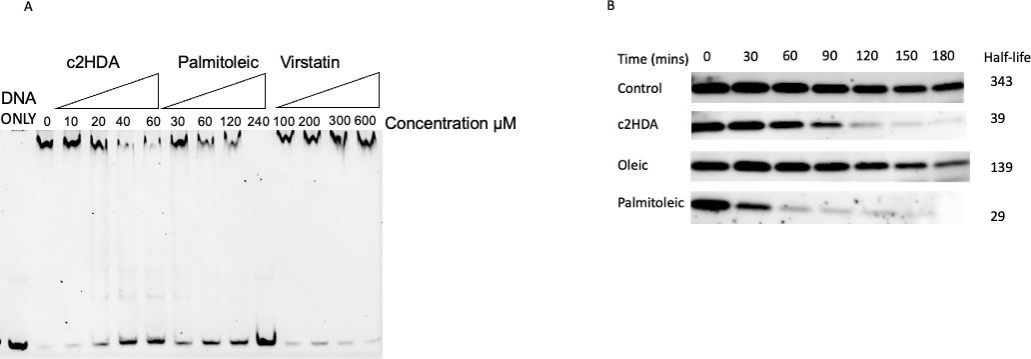
c2HDA impairs the functions of ToxT and targets it for degradation. (**A**) c2HDA interacts with ToxT, preventing it from binding to its target promoters. ToxT protein (3 µM) was mixed with varying concentrations of c2HDA, palmitoleic acid, and virstatin as indicated. Ability of the chemicals to prevent ToxT from binding to the *tcpA-F* promoter was assessed using EMSA. (**B**) c2HDA interacts with ToxT and targets it for degradation. ToxT levels were monitored by western blot for cultures grown in the presence of c2HDA (20 µM), oleic acid (30 µM), and palmitoleic acid (40 µM), respectively. Samples were taken every 30 min for 3 h and the half-life of ToxT was calculated.

We next sought to determine the mechanism through which c2HDA modulates ToxT function. We hypothesized that c2HDA inhibits ToxT from binding to its target promoters, as has been reported for other related long-chain unsaturated fatty acids (11, 13). To examine this directly, we purified ToxT protein and tested its ability to interact with *tcpA-F* promoter DNA in the presence of increasing concentrations of c2HDA using the electrophoretic mobility shift assay (EMSA). c2HDA efficiently prevented ToxT from binding to the *tcpA-F* promoter at a lower concentration (40 µM) than was required for palmitoleic acid (120 µM), whereas the small molecule virstatin (previously reported to interact with ToxT) did not inhibit ToxT at the highest concentration tested (600 µM) (Fig. 6A). These data demonstrate the high efficacy of c2HDA to disrupt the ability of ToxT to bind its target promoters.

We next sought to determine the effects of c2HDA on ToxT stability. It was previously reported that ToxT function is terminated by proteolysis of the ToxT protein, preventing the positive auto-transcription loop of *tcpA-F* and *toxT* (41, 43). We hypothesized that c2HDA prevents ToxT functions and renders it more susceptible to proteolysis, as has been shown for similar transcriptional regulators (31, 44). To test this hypothesis, we determined the effects of c2HDA on the half-life of ToxT. Cultures were grown under CT-inducing conditions and treated with 20 µM c2HDA or physiologically relevant concentrations of oleic and palmitoleic acids (40 µM, see above), respectively. Transcription and translation were halted by adding a cocktail of antibiotics, and samples were taken every 30 min for 3 h. We utilized a chromosomal Δ*toxT* mutant carrying a plasmid-borne construct with a His-tagged ToxT and quantified this protein by western blotting (Fig. 6B). c2HDA and palmitoleic acid reduced the half-life of ToxT from 343 minutes in the untreated control to 39 min and 29 min, respectively (Fig. 6B), suggesting that these chemicals mediate accelerated ToxT degradation. It is therefore conceivable that fatty acids act to repress ToxT through multiple mechanisms that are not mutually exclusive. However, due to structural differences, chemicals may show varying efficiency with multiple mechanisms. Indeed, the small molecule Virstatin was shown to repress ToxT by primarily disrupting dimerization and was thus unable to do so in instances where dimerization was not necessary for ToxT activity (15). Thus, c2HDA targets ToxT-mediated virulence by functional inhibition and consequent clearance of ToxT protein.

### c2HDA acts as a potent allosteric regulator of ToxT

**Fig 7:**
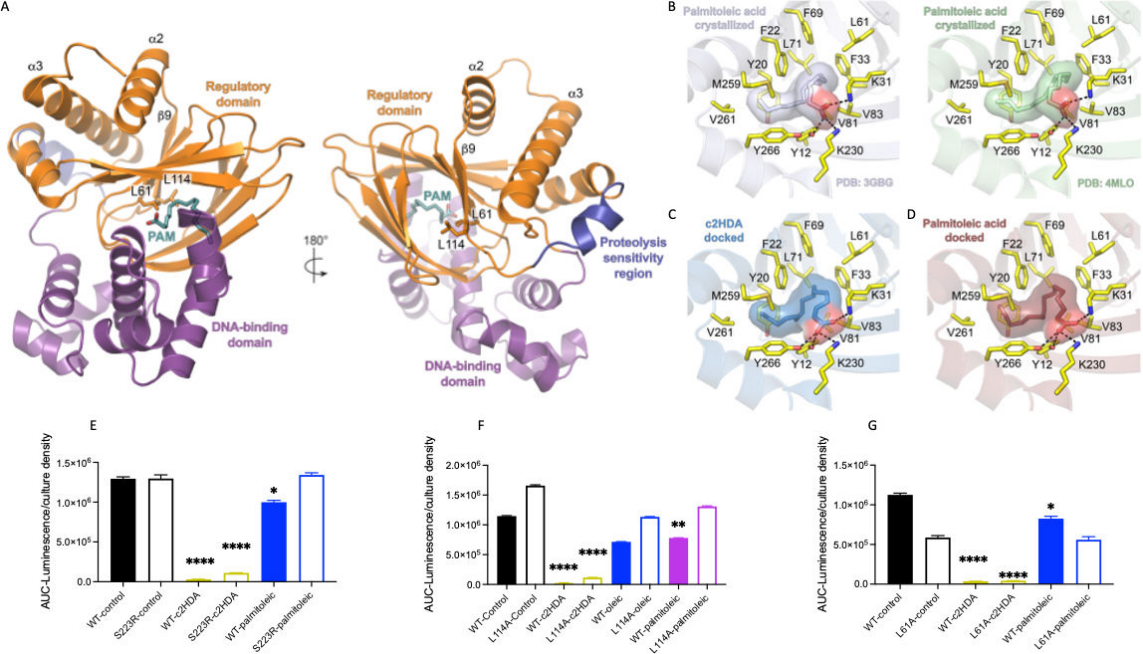
c2HDA acts as a potent allosteric regulator of ToxT. (**A**) Crystallized structure of the ToxT monomer (PDB: 4MLO) is shown in two orientations with the N-terminal regulatory domain colored orange and the C-terminal DNA binding domains colored purple. The proteolysis sensitivity region (residues 100–110) is colored dark blue. A co-crystallized palmitoleic acid molecule (PAM, teal) marks the relative position of the fatty acid binding pocket. The position of the L61 and L114 side chains is shown, and the a2 and a3 dimerization helices are labeled. (**B**) Zoomed views of the fatty acid binding pocket in ToxT monomers co-crystallized with palmitoleic acid (left: PDB: 3GBG, light blue; right: PDB: 4MLO, green). Side chains lining the pocket are colored yellow and labeled. Black dashes denote hydrogen bonds. (**C and D**) Computational docking of c2HDA (**C**, sky blue) and palmitoleic acid (**D**, raspberry) into the crystallized ToxT structure (PDB: 3GBG). Side chains lining the pocket are colored yellow and labeled. Black dashes indicate predicted hydrogen bonds. Docking was performed using AutoDock 2.4 without a defined/restricted binding region. Each model depicts the predicted ligand conformation with the lowest free energy of binding (−7.03 and −7.13 kcal/mol for c2HDA and palmitoleic acid, respectively). (**E through G**) c2HDA potently represses destabilizing ToxT mutants that alleviate the inhibitory effects of other fatty acids. Δ*toxT* strains carrying a *lacZ::_ctxAB_-luxCDABE* reporter fusion and an additional plasmid expressing either wildtype or mutant (S223R [**E**]; L114A [**F**]; L61A [**G**]) ToxT were cultured in AKI medium and supplemented with either 20 µM of c2HDA, 30 µM oleic acid, or 40 µM palmitoleic acid as indicated. Expression of *ctxAB* is presented as the area under the curve of luminescence normalized to culture density. Error bars represent standard deviations of 5 replicates.

ToxT contains an N-terminal regulatory domain that mediates dimerization and a C-terminal DNA-binding domain (12) (Fig. 7A). Structural studies suggest that the configuration of these domains is allosterically controlled by fatty acid binding, which traps ToxT monomers in a conformation that is not compatible with dimerization and proper assembly on promoter DNA (13). Release of the fatty acid is thought to relax the structure, allowing the a2 and a3 helices to adopt a conformation that permits dimerization (13). In co-crystallized complexes, palmitoleic acid binds ToxT in an extensive hydrophobic pocket lined with residues from both the N- and C-terminal domains and is further stabilized through hydrogen bonding with Y12, K31, and K230, which together anchor the carboxylate head (13, 32, 45) (Fig. 7B). L61 lies at the edge of this pocket where it forms hydrophobic interactions with the bound fatty acid on one side and L114 on the other (Fig. 7A and B). L114 resides at the base of the b9 strand, which directly connects to a2 (Fig. 7A). L61 and L114 thus serve as critical contacts that mediate conformational coupling between the fatty acid binding pocket and the a2 and a3 helices forming the ToxT dimer interface (13).

To determine if these molecular interactions also contribute to c2HDA’s potent repressive effects, we computationally modeled c2HDA binding to ToxT using an unrestrained fitting procedure in AutoDock 2.4 (46). c2HDA preferentially docks into ToxT’s hydrophobic pocket with the carboxylate head group rotated, where it forms an additional hydrogen bond with Y266 (Fig. 7C). This minor positional shift may be a consequence of the docking algorithm as palmitoleic acid binds with a similar preferential pose and comparable free energy (−7.13 kcal/mol versus −7.03 kcal/mol for c2HDA) when fit using the same parametrization (Fig. 7D).

Our molecular docking predicts c2HDA may tightly restrain ToxT, locking it in a conformation that prohibits dimerization and impairs favorable association with promoter DNA. We hypothesized that perturbations to the fatty acid pocket and/or conformational coupling would reduce c2HDA’s repressive effects, as has been observed for other fatty acids. To test this, we first analyzed the S223R mutation, which indirectly destabilizes fatty acid binding by sterically forcing ToxT’s N- and C-terminal domains apart (12). We anticipated that c2HDA would lose its inhibitory effects on ToxT^S223R^ if this chemical interacted in the same way as reported for palmitoleic acid (12). While we observed reduced efficacy, 2 µM c2HDA still strongly inhibited reporter gene expression by 3-fold with ToxT^S223R^ as compared to a 4-fold reduction observed with wild-type ToxT (Fig. 7E). Next, we tested whether mutations disrupting the physical connections that coordinate conformational coupling (L114A and L61A) could alter the repressive effects of c2HDA and palmitoleic acid. L114A previously showed enhanced regulatory domain interactions in the bacterial-two-hybrid assay, which correlated with reduced sensitivity to various long-chain fatty acids and the small molecule virstatin (12). c2HDA repressed *ctxAB* in the ToxT^L114A^ strain, whereas both palmitoleic and oleic acid elicited weaker repressive effects compared with the wild-type as expected for this mutant (Fig. 7F). The ToxT^L61A^ mutant induced lower *ctxAB* expression compared with the wild-type but was sufficient to evaluate the repressive effects of the fatty acids. We found that palmitoleic was no longer inhibitory to ToxT^L61A^, whereas c2HDA strongly repressed reporter gene expression in the context of this mutant (Fig. 7G). Finally, we individually mutated the F33 and Y12 side chains that lie on opposite sides of the fatty acid binding pocket (Fig. 7B and C) to alanine and tested whether these substitutions altered the repressive effects of c2HDA and palmitoleic acid. c2HDA similarly showed strong repression of the reporter readout for these binding pocket mutations (Fig. S4). These data suggest that c2HDA binds to ToxT’s fatty acid pocket with high affinity and that this interaction is likely sufficient to maintain a restrained conformation that represses its activity even when destabilizing mutations are also present.

## DISCUSSION

We report that *V. cholerae* integrates interspecies DSFs, normally employed as quorum-sensing signals by members of the proteobacteria, to regulate virulence. The most potent of these was c2HDA. This signal targets the central transcriptional regulator of virulence (ToxT), effectively inhibiting its functions and consequently attenuating CT production and synthesis of the toxin-co-regulated pilus (Fig. 2B and 3). Unlike other related long-chain fatty acids that have been reported to repress *V. cholerae* virulence (9–11, 13), DSFs are a rare class of cis-2 unsaturated fatty acids. We found that chemical structure and functional groups dictate the potency of fatty-acid signals in repressing ToxT-mediated virulence-gene expression, and c2HDA possesses the optimal structure. Thus, c2HDA acts as a precise chemical signal with profound effects in regulating important virulence factors for *V. cholerae* colonization.

Although first discovered in the plant pathogen *Xanthomonas campestris* pv. campestris, increasing evidence shows that DSFs contribute to the regulation of important virulence factors, biofilm formation, and motility in a wide array of plant, animal, and human pathogens (19, 20). DSF synthesis is dependent upon *rpfF*, which is not present in *V. cholerae*. Therefore, in the gut, *V. cholerae* likely integrates DSFs originating from organisms producing these chemicals in the gut environment. Reports have indicated the presence of DSF producers such as *Stenotrophomonas maltophilia* in the crypts of both human and murine large intestines (47, 48). Thus, it is likely that *V. cholerae* senses these chemicals produced by microbiota to repress important virulence factors through a form of interspecies communication. Fatty acids, such as oleic acid, exist in high abundance in the upper intestinal tract. As DSF-producing microbiota are present in the lower intestinal tract, we propose a model in which *V. cholera* utilizes the different classes of these chemicals as signals to modulate virulence. In the ileal lumen, *V. cholerae* may use oleic acid and other related repressive fatty acids that exist in high concentrations to ensure that the energy-intensive virulence factors are produced only at permissible intestinal sites such as the mucus layer. Once *V. cholerae* has colonized the mucus layer and micro-colonies have formed, signals present in the crypts, such as the DSFs, may play an important role in effectively downregulating virulence in anticipation of environmental phase and dissemination. This regulatory mechanism is not restricted to *V. cholerae* only. We have previously shown that other enteric pathogens such as *Salmonella* and *Shigella* sense c2HDA and other long-chain fatty acids using AraC-type transcriptional regulators to regulate virulence and colonization (31, 44, 49–51). Thus, DSF-signaling is a common mechanism that several enteric pathogens employ to regulate pathogenicity and survival in the gut.

In the canonical DSF-signaling systems, DSFs are sensed by either a two-component system like RpfC/RpfG or multidomain proteins that contain PAS, GGGEF, and EAL modules, both of which mechanisms regulate cyclic di-GMP turnover (17, 18, 23, 27). These systems have not been reported in *V. cholerae* thus far. *V. cholerae* instead integrates these signals through the central transcriptional regulator ToxT to control its virulence. Here, we found that c2HDA efficiently represses ToxT-dependent virulence with greater potency relative to other long-chain fatty acids lacking the characteristic cis-2 unsaturation. Our data argue that c2HDA interferes with ToxT-mediated virulence by functional inhibition and consequent clearance of the ToxT protein. Molecular docking suggests that c2HDA binds ToxT within the same hydrophobic pocket that is targeted by other fatty acids. We find, however, that c2HDA can still repress destabilizing ToxT mutants that alleviate the inhibitory effects of other compounds like palmitoleic acid. Based on these observations, we speculate that c2HDA’s increased efficacy arises in part from its apparent higher affinity in binding ToxT, which could more effectively keep ToxT monomers in a restrained conformation that ultimately reduces dimerization and impairs favorable association with promoter DNA. Our data do not rule out that c2HDA can also exploit other alternative binding modes or sites to allosterically regulate ToxT in a manner distinct from other fatty acids and that these mechanisms of control may not be mutually exclusive. We note that although c2HDA is more efficient than palmitoleic acid at repressing ToxT-mediated transcription of virulence genes *in vivo* and impairing binding to promoter DNA *in vitro*, both chemicals promote the degradation of ToxT *in vivo*. It was previously shown that mutations within the region spanning residues 100–110 (Fig. 7A, dark blue) alter ToxT sensitivity to proteolysis and its response to various effectors including bile, linoleic acid, and bicarbonate (52). c2HDA and palmitoleic acid may differentially affect this proteolysis sensitivity region when they interact with ToxT but in a manner that is not directly tied to the allosteric control of dimerization. Further biochemical and structural studies will be necessary to investigate these possibilities in greater detail.

Overall, we describe a potent and precise signal regulating *V. cholerae* virulence and other enteric pathogens as previously reported. This signal is indicative of the intrinsic interaction between these pathogens and the microbiota to optimize virulence and survival. As this signal displays profound efficacy, this interaction may provide an opportunity to design therapeutics targeting this regulatory pathway.

## MATERIAL AND METHODS

### Strains and culture conditions

*V. cholerae* N16961 EI Tor strain and its derivatives were used for all the experiments (Table S1). Where indicated, *V. cholerae* strain C6706 was used. The strain was grown in LB medium (containing 5 g NaCl, 5 g yeast extract, 10 g tryptone, and 15 g agar per liter). Antibiotics were added at the following concentrations where necessary: 200 µg/mL streptomycin, 20 µg/mL chloramphenicol, and 75 µg/mL kanamycin. For luciferase assays and cholera toxin quantification, cultures were grown in AKI medium (containing 5 g NaCl, 3 g NaHCO,_3_ 4 g yeast extract, and 15 g peptone per liter) as previously described (53). Sodium bicarbonate was freshly prepared.

### Plasmids and strain construction

Reporter-gene fusions and protein expression plasmids were constructed using the Gibson assembly method (54). Gene deletion mutants were generated by homologous recombination (55). The deletion cassette was constructed by cloning 700 nucleotide gene-flanking regions into pCVD422. Plasmids were first transformed into *Escherichia coli* DH5alpha, SM10 λ*pir,* or EC100 λ*pir* before subsequent transfer into *V. cholerae*.

### Site-directed mutagenesis

ToxT site-directed mutants were constructed using the Agilent QuickChange Lightning kit according to the manufacturer’s instructions. *toxT* was cloned into the pEBM1 vector under the control of the trc promoter and used for mutagenesis. All plasmids carrying mutants were sequenced by Plasmisaurius Inc. Plasmids were transformed into *V. cholerae* by electroporation.

### Luciferase assays

Strains carrying a *luxCDABE* reporter fusion either on a plasmid (56) or integrated on the chromosome were cultured overnight in LB. The cultures were washed with PBS, and culture density was measured. Overnight cultures were used to inoculate AKI medium at a starting OD_600_ of 0.02. Fatty acids and antibiotics were added as necessary, and the assay was conducted at 37°C. Luminescence was measured in a BioTek Synergy H1 microplate reader.

### Cholera toxin and the toxin co-regulated pilus quantification

For cholera toxin quantification, cultures were grown in AKI medium supplemented with the chemicals to be tested. Cultures were grown standing for 4 h and then shaking for 4 h. Samples were taken and centrifuged to separate supernatants and bacterial pellets. Bacterial pellets were lysed in the protein lysis buffer (containing 50 mM Na_2_H_2_PO_4_ and 500 mM NaCl) supplemented with 1 mM PMSF, 40 ng/mL lysozyme, and 10 ng/mL DNase I. Lysis was performed by freeze-thawing the sample for a total of three cycles. Supernatants were concentrated with Amicon Ultra Filter (Millipore). The total protein concentration was determined using the Bradford Assay according to the manufacturer’s instructions (Bio-Rad). Equal amounts of protein for all samples were mixed with 4× loading dye, and the samples were denatured by heat at 95°C for 5 min. Samples were separated on 12% Bis-Tris gels (NUPAGE; Life Technologies). Western blotting was performed using the rabbit polyclonal anti-cholera toxin (Sigma Aldrich) diluted 1:2,000 as the primary antibody and the goat anti-rabbit IgG horseradish peroxidase-conjugated antibody (Thermo Scientific) diluted 1:10,000 as the secondary antibody.

For toxin co-regulated pilus production, cultures were grown in LB with a starting pH of 6.5 standing for 16 h. Optical density was measured as an indicator of agglutination mediated by the toxin-coregulated pilus.

### Quantitative PCR

The wild-type strain was cultured in AKI medium with chemicals. RNA was extracted using the TRIzol reagent according to the manufacturer’s instructions. Briefly, 0.25 mL of bacterial sample was mixed with 1 mL of TRIzol. The sample was then lysed by bead-beating in 2 mL glass bead-preloaded microtubes (Omni International). Samples were centrifuged to separate the beads. The supernatant was mixed with 0.2 mL of chloroform, incubated for 3 min, and then centrifuged. The RNA sample was precipitated using isopropanol and washed twice with 75% ethanol. The dried sample was reconstituted in RNase-free water. qPCR was performed using the iTaq Universal SYBR Green One-step kit (Bio-Rad) according to the manufacturer’s instructions. For each sample, 100 ng of RNA was used, and experiments were performed with three technical replicates. Reverse transcription was performed at 50°C for 10 min, and the cDNA was initially denatured for 5 min at 95°C. Quantitative PCR was performed using specific primers for the genes to be tested. *rpoB* was reverse transcribed and amplified for normalization.

### Half-life assay

To determine the ToxT half-life, we utilized a ∆*toxT* mutant carrying a plasmid-borne construct of *toxT* under an IPTG-inducible promoter with a C-terminal His-tag. An overnight culture was used to inoculate AKI medium containing the chemicals to be tested. The cultures were induced with IPTG for 3 h. OD_600_ was adjusted to 1 for all the cultures. A cocktail of antibiotics was added to halt transcription and translation. Cultures were incubated at 37°C with shaking, and the samples were taken every 30 min for western blotting. Western blotting was performed using a mouse anti-His-tag antibody (diluted 1:2,000; Thermo Scientific) and an anti-mouse IgG secondary antibody (1:10,000; Thermo Scientific). Half-life was calculated as the difference in density between time point zero and the last signal time point, as previously described (57).

### ToxT expression and purification

*toxT* was cloned into pCAV4, a modified T7 expression vector that introduces an N-terminal 6×His-NusA tag, followed by a human rhinovirus (HRV) 3C protease site upstream of the ToxT coding sequence. The construct was initially transformed into *E. coli* DH5alpha to confirm the cloning and then transferred into *E. coli* BL21(DE3). The strain was induced overnight at 19°C with 0.3 mM isopropyl-β-d-thiogalactopyranoside (IPTG). Cells were centrifuged and resuspended in nickel buffer (20  mM HEPES [pH 7.5], 500  mM NaCl, 5% glycerol, 30  mM imidazole, and 5  mM β-mercaptoethanol). Cells were centrifuged, and the supernatant was applied to a 5 mL HiTrap chelating column charged with nickel sulfate. After washing with nickel buffer, the protein was eluted with an imidazole gradient (30 mM to 500 mM). Pooled elution fractions were dialyzed into heparin buffer (20  mM HEPES [pH 7.5], 300  mM NaCl, 1  mM EDTA, 5% glycerol, and 1  mM dithiothreitol [DTT]) in the presence of HRV 3C protease to cleave the 6xHis-NusA tag and then applied to a 5 mL heparin HiTrap heparin column. The column was washed with heparin buffer, and the protein was eluted with a gradient of NaCl (150 mM to 1 M). Pooled fractions containing the ToxT protein were concentrated and injected into a Superdex 200 10/300 sizing column. Purified ToxT was stored in buffer containing 20  mM HEPES [pH 7.3], 150  mM KCl, and 1  mM DTT.

### Electrophoretic mobility shift assays

EMSAs were performed as previously described (44). Briefly, 10 nM of the *tcpA-F* were mixed with varying concentrations of ToxT protein. Chemicals to be tested were added at different concentrations. Binding was performed at room temperature for 30 min. Samples were separated on 6% Novex Tris-borate-EDTA (TBE) DNA retardation gels (Thermo Fisher Scientific), and DNA was stained using SYBR green (Invitrogen).

### Statistical analysis

Results are presented as means with standard deviations. Comparisons between controls and tests were evaluated using Student’s *t*-tests.

## SUPPLEMENTAL MATERIAL

Supplemental material mbio.01537-25-s0001.docx Supplemental figures and table.

ASM does not own the copyrights to Supplemental Material that may be linked to, or accessed through, an article. The authors have granted ASM a non-exclusive, world-wide license to publish the Supplemental Material files. Please contact the corresponding author directly for reuse.
