# Supplementary material for: Vibrio cholerae integrates interspecies quorum-sensing signals to regulate virulence
Source: mBio. 2025 Jul 31;16(9):e01537-25. doi: 10.1128/mbio.01537-25 (PMC12421807; doi:10.1128/mbio.01537-25)
Supplement: Supplemental material — Supplemental figures and table. [file mbio.01537-25-s0001.docx]

**Supplementary Information**

**Supplementary Table 1: Strains and plasmids**

| **Strain/plasmid** | **Genotype/sequence** | **Reference** |
| --- | --- | --- |
| CA5671 | *V. cholerae,* ∆*toxT* | This study |
| CA5587 | *V. cholerae,* ∆*toxT, lacZ*::*_ctxAB_-luxCDABE* | This study |
| CA5588 | *V. cholerae, lacZ*::*_ctxAB_-luxCDABE* | This study |
| CA5823 | *C. sakazakii*, wild type |  |
| CA5882 | *C. sakazakii, ∆rpfF* | This study |
| CA6058 | *C. sakazakii, ∆rpfF*, pFAB | This study |
| CA5883 | *C. sakazakii, ∆rpfF*, pFAB-*rpfF* | This study |
| **Plasmids** |  |  |
| CA5671 | p*toxT*-SUMO | This study |
| CA5689 | p*toxT*-SUMO-ToxTL61A | This study |
| CA5691 | p*toxT*-SUMO-ToxTK31A | This study |
| CA5686 | p*toxT*-SUMO-ToxTV83A | This study |
| CA5722 | p*toxT*-SUMO-ToxTY12A | This study |
| CA5727 | p*toxT*-SUMO-ToxTL114A | This study |
| CA5724 | p*toxT*-SUMO-ToxTL114A | This study |
| CA5735 | p*toxT*-SUMO-ToxTS223R | This study |
| CA5961 | p*_tcpA-F_-luxCDABE* | This study |
| CA5198 | pBRR-lux | [[57](#_ENREF_57)] |
| CA5128 | pTD101 | [[58](#_ENREF_58)] |
| CA5127 | pJL1 | [[58](#_ENREF_58)] |

**Supplementary Figures**

**Supplementary figure 1**

**
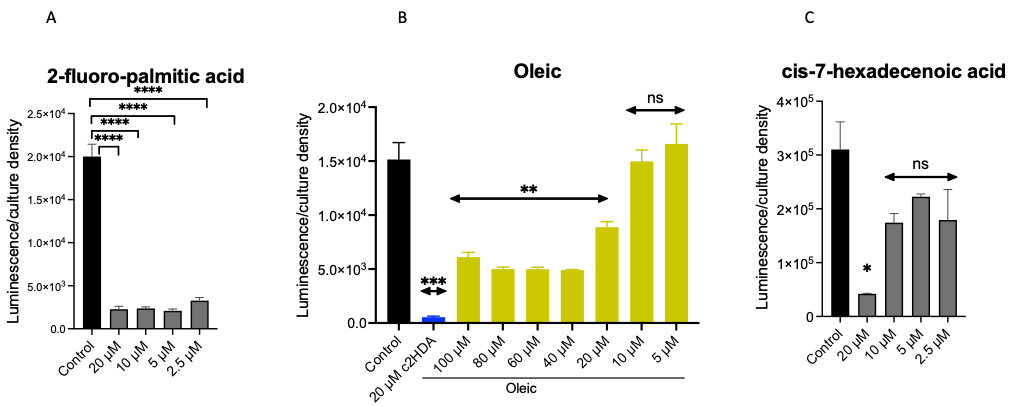
**

**Supplementary Figure 1: Dose-dependent response of selected fatty acids.** A strain carrying a *lacZ*::*_ctxAB_-luxCDABE* construct was cultured under cholera toxin-inducing conditions in the presence of fatty acids. A) 2-fluro-palmitic acid, B) oleic acid, C) cis-7-hexadecenoic acid. Error bars represent standard deviations of 5 replicates. Asterisks denote significant differences from the untreated control (**-P<0.01, *-P<0.05).

Supplementary Figure 2


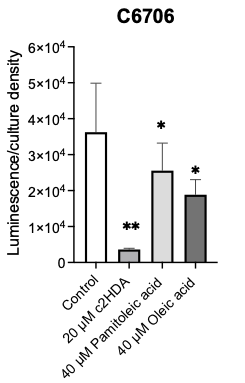


**Supplementary Figure 2: c2HDA represses CT expression in *V. cholerae* C6706 strain.** Strain C6706 carrying a *lacZ*::*_ctxAB_-luxCDABE* construct was cultured under cholera toxin-inducing conditions in the presence of fatty acids. Expression of the reporter fusion is presented as peak luminescence normalized to culture density. Error bars represent standard deviations of 5 replicates. Asterisks denote significant differences from the untreated control (**-P<0.01, *-P<0.05).

**Supplementary Figure 3**

**
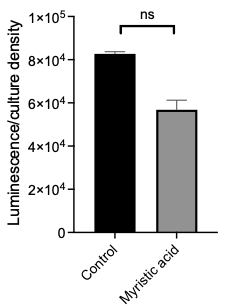
**

**Supplementary Figure 3: Degradation products of c2HDA are not effective in repressing virulence-gene expression.** Strain carrying a *lacZ*::*_ctxAB_-luxCDABE* construct was cultured under cholera toxin-inducing conditions in the presence of myristic acid. Expression of the reporter fusion is presented as luminescence normalized to culture density. Error bars represent standard deviations of 5 replicates.

Supplementary Figure 4


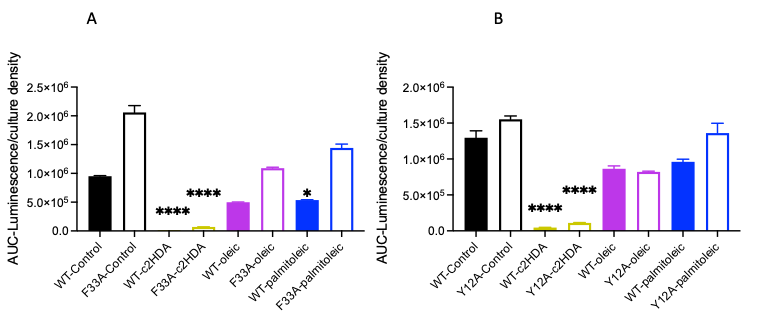


**Supplementary Figure 4: c2HDA represses ToxT amino acid mutants predicted to play a role in binding to this signal.** A Δ*toxT* strain carrying a *ctxAB→luxCDABE* reporter fusion and ToxT expressed from a plasmid was cultured in AKI medium and supplemented with the chemical signals as indicated. A) ToxTF33A and B) Y12A. Expression of *ctxAB* is presented as area under curve of luminescence normalized to culture density.
